# PINCER (A Platform Study for solId orgaN CancERs): an agile pan-network platform study to deliver high-quality translational research

**DOI:** 10.1093/bjs/znad097

**Published:** 2023-04-20

**Authors:** Robert P Jones, Ainhoa Mielgo, Michael Schmid, Danielle Bury, Timothy Andrews, Susanne Burdak-Rothkamm, Michael Shackcloth, Timothy J. S. Cross, Stephen Fenwick, Hassan Z Malik, Rafa Diaz-Nieto, Christian Ottensmeier, Daniel H Palmer, Dale Vimalachandran

**Affiliations:** Department of Molecular and Clinical Cancer Medicine, Institute of Systems, Molecular and Integrative Biology, University of Liverpool, Liverpool, UK; Department of Hepatobiliary Surgery, Liverpool University Teaching Hospitals NHS Foundation Trust, Liverpool, UK; Department of Molecular and Clinical Cancer Medicine, Institute of Systems, Molecular and Integrative Biology, University of Liverpool, Liverpool, UK; Department of Molecular and Clinical Cancer Medicine, Institute of Systems, Molecular and Integrative Biology, University of Liverpool, Liverpool, UK; Department of Pathology, Blackpool Teaching Hospitals NHS Foundation Trust, Blackpool, UK; Department of Molecular and Clinical Cancer Medicine, Institute of Systems, Molecular and Integrative Biology, University of Liverpool, Liverpool, UK; Department of Cellular Pathology, Liverpool University Teaching Hospitals NHS Foundation Trust, Liverpool, UK; Department of Molecular and Clinical Cancer Medicine, Institute of Systems, Molecular and Integrative Biology, University of Liverpool, Liverpool, UK; Department of Cellular Pathology, Liverpool University Teaching Hospitals NHS Foundation Trust, Liverpool, UK; Department of Thoracic Surgery, Liverpool Heart and Chest Hospital, Liverpool, UK; Department of Hepatology, Liverpool University Teaching Hospitals NHS Foundation Trust, Liverpool, UK; Department of Hepatobiliary Surgery, Liverpool University Teaching Hospitals NHS Foundation Trust, Liverpool, UK; Department of Hepatobiliary Surgery, Liverpool University Teaching Hospitals NHS Foundation Trust, Liverpool, UK; Department of Hepatobiliary Surgery, Liverpool University Teaching Hospitals NHS Foundation Trust, Liverpool, UK; Department of Molecular and Clinical Cancer Medicine, Institute of Systems, Molecular and Integrative Biology, University of Liverpool, Liverpool, UK; Department of Oncology, Clatterbridge Cancer Centre, Liverpool, UK; Department of Molecular and Clinical Cancer Medicine, Institute of Systems, Molecular and Integrative Biology, University of Liverpool, Liverpool, UK; Department of Oncology, Clatterbridge Cancer Centre, Liverpool, UK; Department of Molecular and Clinical Cancer Medicine, Institute of Systems, Molecular and Integrative Biology, University of Liverpool, Liverpool, UK; Department of Colorectal Surgery, Countess of Chester NHS Foundation Trust, Chester, UK

## Background

Translational research is the crucial link between basic science discovery and endeavours to improve the human condition through improved healthcare diagnostics and interventions^1,2^. Basic science often relies on cell lines and animal models, which, although attractive for their stability and reproducibility, are often limited in their external validity to humans. There is also a desire to limit animal testing by producing human-derived models^3^. However, scientists are often limited by their ability to access relevant human samples.

**Fig. 1:**
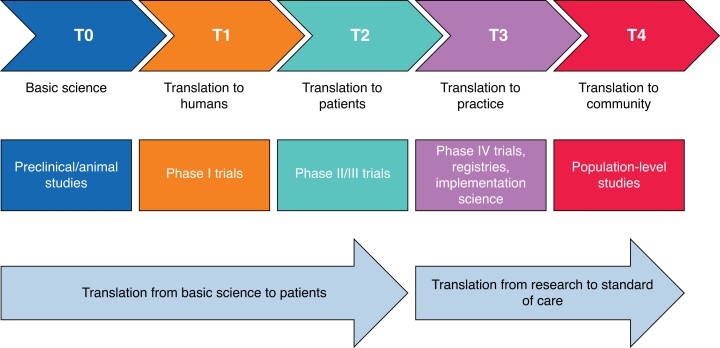
Broad domains of translational research Cancer surgeons are ideally placed to deliver stages T1–T3.

Cancer surgeons are ideal translational researchers and collaborators^4^. They are working with enthused and motivated groups of patients who are often keen to engage in research. They are also acutely aware of the clinical unknowns facing patients and doctors managing cancer, making them well placed to guide basic science into translational research (*Fig. 1*). Traditionally, this work has been the preserve of clinicians working in established academic centres with existing research networks. However, much cancer treatment takes place outside of these academic centres. Interventions often take place in multiple geographical locations as patients progress through multimodal treatment. Allowing research to follow patients to where they are treated is essential, not only to allow clinically relevant research and sampling but also to ensure equity of access^5^.

Current research and development processes are often impractical for such multisite collaborations; smaller translational studies frequently rely on existing laboratory resource rather than having explicit funding awards, and organizations may be reluctant to sponsor small studies without income. These studies are also burdensome to open, with hospitals requiring similar levels of governance for an interventional drug trial as a small observational cohort. This burden is often duplicated for every location.

To address these issues, the authors have developed an agile regional platform study to deliver high-quality translational research across a cancer network with two key objectives: to allow longitudinal sampling of patients with cancer across multiple treatment sites in different organizations; and to support the delivery of new translational research collaborations at existing sites.

## Methods

**Fig. 2:**
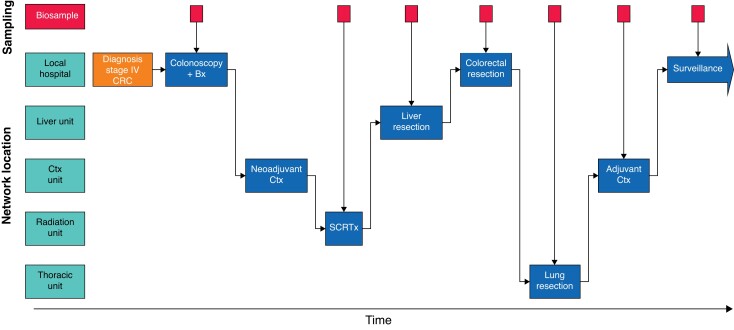
Example of how a patient with stage IV colorectal cancer may receive multiple treatments at different geographical locations Consent for PINCER means that multiple samples and data points can be collected as the patient progresses through treatments at these sites. As well as tissue and blood, radiological and quality-of-life data can be collected to provide a comprehensive research resource. CRC, colorectal cancer; Bx, biopsy; Ctx, chemotherapy; SCRTx, short-course radiotherapy.

PINCER is a regional platform study designed to allow efficient patient recruitment for translational research (protocol available in *Appendix S1*) across multiple geographical sites within a cancer network (*Fig. 2*). This allows longitudinal translational cancer research with sampling at multiple time points, irrespective of where the patient is being treated.

The study has full ethical approval (NW REC 15/NW/0477), and is registered on the UK National Institute for Health Research (NIHR) National Research Portfolio (CPMS 46169), and at ClinicalTrials.gov (NCT05650125). The platform has been designed to minimize burden, and can be delivered with little ongoing research and development support. This model provides resilience in the face of competing studies which may be perceived as having higher financial or clinical value by organizations (for example COVID-19 studies, commercial clinical trials), meaning that it can be delivered without relying on additional staffing support.

The flexible protocol allows sampling of surgically resected tumour and adjacent normal tissue taken as part of standard of care, with additional passes of biopsy where patients are having a diagnostic biopsy. Blood samples and quality-of-life questionnaires can be requested up to 12 months after initial sampling. Matched pseudoanonymized clinical, pathological, and radiological data (including clinical images) can also be accessed for all patients. The platform can therefore support a wide number of translational research projects (*Table 1*), and is used by surgeons, oncologists, gastroenterologists, and pathologists, among others.

**Table 1:** Selected substudies currently using PINCER platform

Study	Sample types	No. of sites contributing samples	No. of patients
ctDNA in high-risk stage II–III colorectal cancer	Tumour/blood	3	55
Cholangiocarcinoma tissue slices	Bile duct cancers	1	32
Tumour microenvironment in pancreatic liver metastases	Blood and diagnostic liver biopsy	2	24
Tumour microenvironment in metastatic colorectal cancer	Matched liver/lung/primary tissue, bloods	5	26
ctDNA stage IV colorectal cancer	Resected colorectal liver metastasis, longitudinal bloods to 12 months	2	8
Hepatic organoid modelling for assessment of drug efficacy and toxicity	Liver tissue	2	410
Meiotic protein dysfunction in hepatocellular carcinoma	Hepatocellular carcinoma and background liver biopsy in high-risk patients with cirrhosis	4	8

ctDNA, circulating tumour DNA.

### Model

Using a central sponsorship model provided by the University of Liverpool, seven hospitals (including academic centres and regional hospitals) across a regional cancer alliance network have been added to the PINCER platform with local research and development approval at each site. Each site has a traditional principal investigator (PI), with a trainee clinician registered as an associate PI using the NIHR Associate PI scheme^6^. This ensures that trainees are indoctrinated in a culture of research as standard of care, developing engaged and independent researchers of the future who know how to interact and collaborate with scientists, and together enable the translation of new laboratory discoveries into the clinics.

All patients being treated for solid organ cancer under the oversight of a cancer multidisciplinary team are eligible for recruitment. Patients are given a patient information sheet in advance, usually in an outpatient setting. They are then approached and consented on the day of first treatment. A clearly defined standard operating protocol (SOP) for tissue sampling has been developed with pathologists to ensure that routine histopathological assessment of margins, etc. is not compromised (*Appendix S2*). Samples are transferred to collaborators using an overarching organization information document and sample log. There is no tissue sampling or storage of samples at any hospital site, meaning that there are no excess costs to the host organization. Outputs do not require acknowledgement of any individuals on the PINCER committee, but acknowledgement of the platform’s role in delivering biosamples is expected.

### Governance

In contrast to a tissue bank, PINCER allows sampling only for a predefined and approved research question (*Table 1*). Collaborations using the platform can be with local, regional, national, or international groups. These collaborators can be academic or commercial, and a per-sample fee or similar can be negotiated on a project-by-project basis to develop pump priming funding to support other translational studies. Commercial collaborations are explicitly described in the consent and patient information sheets, and contractual negotiations with commercial partners are managed by the sponsor.

Each substudy planning to use the platform applies to the PINCER steering committee. This committee of clinicians and academics assesses the quality of the research question, and ensures that the proposal and sampling are in keeping with the overarching ethics approval (*Appendix S1*). Individual ethical approval for each substudy is not required. For each substudy, researchers are expected to be certified with NIHR Good Clinical Practice and Human Tissue Authority (HTA) sample storage regulations. Each substudy provides its own sampling SOP which defines the patient group, number of patients, samples and storage/transfer arrangements, and predicted outputs. Each substudy investigator tracks patients across sites and liaises with the local team to facilitate sampling, and is responsible for annual tissue storage returns.

### Example substudies

PINCER has been open since December 2018. Recruitment is acceptable to patients, with 558 of 563 screened patients (99.1 per cent) consenting and providing samples. A summary of some current PINCER substudies is included in *Table 1*.

## Discussion

The authors have developed an agile translational research platform that allows clinicians and basic scientists to collaborate efficiently and effectively. While maintaining a high level of research integrity, quality control, and robust governance, PINCER minimizes duplication, meaning that non-academic clinicians can develop studies and recruit and sample patients for high-quality research. PINCER also allows studies to track patients across multiple locations, reflecting standard clinical management, improving access to longitudinal samples and facilitating research access to more patients. PINCER supports pan-specialty research collaborations with additional direct clinical benefits, including closer working and understanding between specialties.

The methodology is easy to embed in routine clinical practice, is acceptable to patients, and requires very limited support from host hospitals. As shown, this methodology delivers a broad number of high-quality translational outputs, and provides an example that can be replicated by other groups that wish to develop academic and commercial research collaborations.

As the platform has evolved, basic scientists have transitioned from passive recipients of samples to active drivers in research, defining the required samples and when they should be taken. Furthermore, basic scientists form a key part of the study governance structure. Although patients are not told on an individual basis about the results of any research on their samples, broader research outputs are distributed via social media and patient research outputs delivered by the NIHR. In the future, the platform could be evolved to allow the delivery of formal cohort trials with embedded translational arms. There is also the potential to assess therapeutics *in vivo*, with longitudinal assessment of established drug response and toxicity in patients with cancer; for example, patients with colorectal liver metastases undergoing resection may have their tumours exposed to standard cytotoxics to assess response while their hepatic tissue is phenotyped to assess the risk of drug-induced liver injury. Although subsequent treatments would not be altered based on these findings, predictions around response and toxicity could be validated when patients are treated with systemic agents for recurrence, providing compelling preliminary data for future trials.

The platform does have limitations. The strictly observational nature of the study means that interventions and window-of-opportunity style studies are not possible, and the additional regulatory oversight necessary for these kinds of research would mean that the platform loses its broad generic strengths, and so has not been pursued. The platform is also limited in interstudy comparisons; the advantage of each substudy defining its own sampling and storage requirements means that direct comparison between translational cohorts can be limited.

In summary, PINCER offers an agile and effective method of delivering high-quality translational research that can be replicated by other groups that wish to pursue a similar approach.

## Supplementary Material

znad097_Supplementary_Data
