# Supplementary material for: PINCER (A Platform Study for solId orgaN CancERs): an agile pan-network platform study to deliver high-quality translational research
Source: Br J Surg. 2023 Apr 20;110(9):1108–11. doi: 10.1093/bjs/znad097 (PMC10805525; doi:10.1093/bjs/znad097)
Supplement: znad097_Supplementary_Data [file znad097_supplementary_data.docx]

**PINCER (A *P*latform Study for sol*I*d orga*N* *C*anc*ER*s): an agile pan-network platform study to deliver high quality translational research**

Robert P. Jones^1,2^, Ainhoa Mielgo^1^, Michael Schmid^1^, Danielle Bury^3^, Timothy Andrews^1,4^, Susanne Burdak-Rothkamm^1,4^, Michael Shackcloth^5^, Timothy JS Cross^6^, Stephen Fenwick^2^, Hassan Z. Malik^2^, Rafa Diaz-Nieto^2^, Christian Ottensmeier^1,7^, Daniel Palmer^1,7^, Dale Vimalachandran^1,8^

1. Department of Molecular & Clinical Cancer Medicine, Institute of Systems, Molecular and Integrative Biology, University of Liverpool, Liverpool, UK
2. Department of Hepatobiliary Surgery, Liverpool University Teaching Hospitals NHS Foundation Trust, Liverpool, UK
3. Department of Pathology, Blackpool Teaching Hospitals NHS Foundation Trust, Blackpool, Lancashire, UK
4. Department of Cellular Pathology, Liverpool University Teaching Hospitals NHS Foundation Trust, Liverpool, UK
5. Department of Thoracic Surgery, Liverpool Heart & Chest Hospital, Liverpool, UK
6. Department of Hepatology, Liverpool University Teaching Hospitals NHS Foundation Trust, Liverpool, UK
7. Department of Oncology, Clatterbridge Cancer Centre, Liverpool, UK
8. Department of Colorectal Surgery, Countess of Chester NHS Foundation Trust, Chester, Cheshire, UK

Corresponding author/address for reprints:

Mr. Robert P Jones

Department of Hepatobiliary Surgery,

Liverpool University Teaching Hospitals NHS Foundation Trust,

Prescot Street,

Liverpool, UK

[robjones@liv.ac.uk](mailto:robjones@liv.ac.uk)

0151 5291234

**Supplementary Materials - Index**

| **Supplementary Appendixes** |  | |
| --- | --- | --- |
| Appendix S1 – PINCER Protocol | *pag. 3* | |
| Appendix S2 – PINCER Tissue Sampling SOP | | *pag. 18* |
|  |  | |
|  |  | |
|  |  | |
|  |  | |
|  |  | |
|  |  | |
|  |  | |
|  |  | |
|  |  | |
|  |  | |
|  |  | |

Appendix S1

**
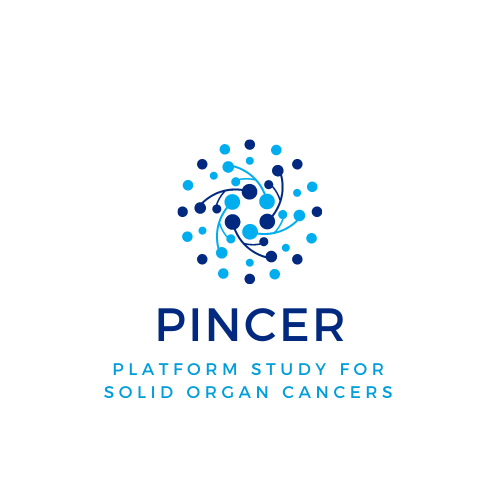
**

Protocol Version 9.0

June 2022

**Study Team**

Chief Investigator: Prof. Daniel Palmer

Study Lead: Mr. Robert Jones

Steering group: Mr. Robert Jones, Mr. Dale Vimalachandran, Dr. Ainhoa Mielgo, Prof. Michael Schmid, Prof. Christian Ottensmeier, Prof. Paula Ghaneh, Prof. Christopher Halloran, Prof. Christopher Goldring, Prof. Daniel Palmer

**Sponsor**

The University of Liverpool is the research Sponsor for this Study. For further information regarding the sponsorship conditions, please contact:

Miss Karen Wilding

Senior Clinical Research Governance Manager

Clinical Directorate

4th Floor Thompson Yates Building

Faculty of Health and Life Sciences

University of Liverpool

Liverpool L69 3GB

T: 07717863747

[sponsor@liverpool.ac.uk](mailto:sponsor@liv.ac.uk)

**STUDY SUMMARY**

This protocol describes the PINCER (A **P**latform Study for sol**I**d orga**N** **C**anc**ER**s) Study and provides information about procedures for entering participants. Every care was taken in its drafting, but corrections or amendments may be necessary. These will be circulated to investigators in the study. Problems relating to this study should be referred, in the first instance, to the Chief Investigator.

This study will adhere to the principles outlined in the NHS Research Governance Framework for Health and Social Care (2nd edition). It will be conducted in compliance with the protocol, the Data Protection Act and other regulatory requirements as appropriate.

**1. INTRODUCTION**

**1.1 BACKGROUND**

Despite improved therapeutic options, the long-term outlook for patients with some solid organ remains dismal (1,2). Surgery is the only potentially curative treatment, but only a minority of patients [10-30%] have technically resectable disease (3). Of those undergoing resection, only around forty percent survive for 5 years (4,5), with the remainder succumbing to disease recurrence (6).

**1.2 RATIONALE FOR CURRENT STUDY**

Existing strategies to predict long-term outcome after treatment of solid organ cancers rely on preoperative radiological staging and post-resection analysis of tumour margin and nodal status (7,8). Improved prognostication through biomarkers has been suggested, and direct analysis of tumour may allow the development of a more personalised therapeutic approach; patients with aggressive disease biology may benefit from adjuvant systemic chemotherapy, more intense follow-up after resection or be better served by non-operative management. Better prediction of long-term outcome would also allow reproducible risk stratification for clinical trials, an important consideration for rare diseases where large RCTs remain difficult. As well as allowing the optimisation of existing treatments, better understanding of key biological pathways involved the pathogenesis of cancers also offers the potential identification of novel therapeutic targets for further investigation. In addition, little is known about how treatment strategies for solid cancers correlate with patient reported outcomes. Numerous qualitative studies and research groups have clearly identified the need to explore patient reported outcomes in a variety of disease groups.

**Generating bespoke biosampling protocols for each individual tumour specific project is onerous, cumbersome and inefficient.** **This study aims to provide a broad platform to maximise the unique access to biopsy and resected tumour specimens available from surgical patients to provide a high-quality and efficient source of biosamples for specific translational projects. Access to historical FFPE samples and clinical data, as well as prospective fresh tissue samples, will allow interrogation of the underlying biology of these cancers. Matched pseudoanonymised clinical and radiological data will allow the development of rich high-yield datasets.**

**2. STUDY DESIGN**

This is a platform tissue collection study to provide biosamples for specific research projects. These sub-studies will be proposed by interested researchers and undergo internal review by the PINCER steering committee (See appendix 1, application form for adoption, and appendix 3, terms of reference for PINCER steering committee). Following approval, each sub study will be delivered on this platform in line with this protocol. Interested researchers will be expected to have full GCP certification and completed MRC HTA training (<https://byglearning.com/mrcrsc-lms/course/index.php?categoryid=1>), and comply with the principals outlined in NIHR Good Clinical Practice guidance.

**This study will not be used to generate biobanks, but to allow biosamples to be used for specific active sub studies.**

***2.1 Study Locations & Approval***

This platform will allow patients having surgery or biopsy for any solid organ cancer to consent to take part in the study. Any site wishing to do this will open as a site for the PINCER Study.

Sub-studies using PINCER will require their own local study lead and SOP written by the local team and this will require approval by the PINCER steering committee. This will be to confirm alignment with the overarching PINCER protocol, define sampling requirements and assess the scientific validity of the study, as well as ensure regulatory oversight (see appendix 3).

***2.2 Fresh Tissue Collection***

Tissue will be obtained either through biopsy (an extra sample of tissue will be taken using the same needle at the time of original biopsy) or after surgical resection (where the tumour tissue would routinely be discarded after sampling for pathological assessment). In the case of tissue retrieved after surgery, a pathologist will ensure that excess tissue removed after resection will not compromise pathological assessment of the resected specimen.

At the same time as biopsy or surgery, a small sample of blood (60ml) may be removed from the venous catheter which the patient has inserted as part of the routine care during their procedure.

After the tissue has been retrieved, tissue and blood will be transported to a HTA approved facility. This will be performed under a locally arranged material transfer agreement (MTA) agreed by the local team between each individual site and the relevant partner organization.

***2.3 Collection of historical FFPE tumour tissue***

Patients who have undergone biopsy or surgery for solid organ cancers will be identified from existing NHS clinical databases which track surgical activity by relevant clinical teams. Their tumour tissue samples will then be identified using local pathology systems, and their archived tissue blocks retrieved. In the case of patients who have had metastatic disease resected, both primary and metastatic tumour tissue will be accessed. A small amount of these tissue blocks will be sampled, and the remnant tissue block returned to the pathology archive. After the tissue has been retrieved, it will be transported to a HTA approved facility. This will be performed under a locally arranged material transfer agreement (MTA) between each individual site and the relevant partner organization.

***2.4 Collection of post-treatment blood samples***

Up to 60mls of blood may be drawn from patients up to 12 months following treatment. After the blood has been retrieved, it will be transported to a HTA approved facility. This will be performed under a locally arranged material transfer agreement (MTA) between each individual site and the relevant partner organization.

***2.5 Quality of Life Analysis***

Where appropriate, patients will be invited to complete EORTC Quality of Life assessment questionnaires up to 12 months following treatment.

***2.6 Pseudoanonymised data & radiology collection***

Linked clinicopathological and radiological data will be retrieved where appropriate and stored on password protected computer systems. Local pseudoanonymisation will be required before these data are shared with other researchers not directly involved in the clinical care of patients.

***2.7 Biosample analysis***

All analyses will be performed in HTA approved laboratories or equivalent. Pseudoanonymised samples may be shared with academic and commercial partner organisations within the UK and abroad, including the USA. All users of biosamples will be expected to have completed MRC HTA training (<https://byglearning.com/mrcrsc-lms/course/index.php?categoryid=1>). Bioanalysis of samples is likely to evolve dependent on the development of new techniques and technologies, but may include some the following techniques:

***Histopathological and radiological analysis***

Samples will undergo routine histopathological analysis using H&E stains. Standard preoperative radiological imaging will be assessed to identify novel radiomic markers that may reflect underlying tumour biology.

***Analysis of fresh tissue***

Genomic analysis will include whole genome and exome sequencing. Transcriptomic and metabolomic analysis will be performed as required. Whole proteome analysis of fresh tissue will be performed using a novel iTRAQ LC-MS/MS based assay, giving high coverage absolute protein quantification. Other cellular analytical techniques will be used as appropriate, for example FACS analysis and geospatial transcriptomic analysis.

***Analysis of FFPE tissue***

Findings in smaller populations of fresh tissue may be validated in larger cohorts of FFPE tissue. Techniques may include IHC, Nanostring, WGS and exome sequencing.

***Analysis of blood and plasma***

After separation of plasma from whole blood, plasma samples will be subject to analysis (including ctDNA quantification).

***Generation of patient derived multi-cellular tumour models***

After the tumour resection has been completed and fresh sample excised, a 5-10mm section may be removed and immediately placed on ice for transport. This piece of excised sample will then undergo enzymatic tissue digestion overnight to generate a single cell suspension for primary cell culture and placed in appropriate media according to published protocols. These patient derived multi-cellular tumour models will then be cultured and characterised for the in-vitro validation of actionable targets identified by the genomic and proteomic analyses. Once individual primary cell models have been characterized and/or treated with cytotoxics they will be cryopreserved and securely stored for future analyses. Those patient derived cell models that are non-viable will be disposed of in accordance with established laboratory codes of practice.

Other scientific techniques may be used depending on specific research questions.

***2.8 Sample Transfer***

Sample transfer from NHS organisations to the University of Liverpool will require a formal Material Transfer Agreement. If an MTA does not already exist, it will be the responsibility of the sub-study lead to organize one before any sample movement.

Sample transfer will be recorded using a formal Sample Transfer Log (see appendix 2). Samples will be transferred from the provider institution direct to the team running the sub-study.

Sample transfer out of, or internally within, the University of Liverpool will be considered by the steering committee on a study-by-study basis (See appendix 3). The lead for each sub-study will contact the steering committee to clarify the need to transfer samples, and request permission to do so. They will be required to demonstrate that the proposed collaboration falls within the context of this study protocol. Permission to release samples will only be given if the proposed collaboration falls within the remit of the protocol, favorable ethical opinion and consent stipulations. Once this permission is given the sub-study applicant will organize, if external to the University of Liverpool, a formal Material Transfer Agreement between the University of Liverpool and the receiving party. This MTA will be shared with the steering committee. Samples will then be tracked using the Sample Transfer Log (see appendix 2). If the movement of samples is internally within the University of Liverpool just the Sample Transfer Log will be completed.

**3. STUDY OBJECTIVES**

Study objectives will be defined for each sub study but may include:

- Identification of potentially important prognostic and predictive genetic, proteomic and transcriptomic changes in solid organ cancers
- Secondary validation screen with preliminary validation of identified prognostic/predictive markers in historical FFPE cancer samples and matched normal tissues
- Development/establishment of novel bioanalytical techniques in solid organ cancers
- Establish patient derived multi-cellular tumour models for in-vitro validation of actionable drug targets and disease modeling
- Assess feasibility of collecting routine quality of life data from patients prior to and following treatment
- Assess patient acceptability of collecting (regular) post treatment blood samples and quality of life assessments

**4. PARTICIPANT ENTRY**

Patients will be identified as eligible for recruitment by the local clinical teams (prospective tissue collection) or from existing clinical datasets (retrospective tissue sampling).

**4.1 INCLUSION CRITERIA**

Solid organ malignancy requiring surgery or biopsy as part of their routine clinical care as decided by the specialist MDT.

**4.2 EXCLUSION CRITERIA**

Refusal or inability to consent

Paediatric patients

**5. STATISTICS AND DATA ANALYSIS**

The overall platform study size (2000 fresh tissue samples, 5000 historical FFPE samples) is based on an estimation of the number of eligible patients who will present through regional MDT meetings and will undergo resection/biopsy during the study period. The 5000 historical samples are based on interrogation of existing clinical datasets. No formal statistical power calculations have been performed. Sample requirements for each sub study will not exceed this and sampling will be performed as per a Standard Operating Procedure (SOP) Document developed by each local team for each sub-study.

**6. REGULATORY ISSUES**

**6.1 ETHICS APPROVAL**

The Chief Investigator has obtained approval from the Research Ethics Committee. The study must be submitted for Site Specific Assessment (SSA) at each participating NHS Trust. The Chief Investigator will require a copy of the Trust R&D approval letter before accepting participants into the study. The study will be conducted in accordance with the recommendations for physicians involved in research on human subjects adopted by the 18th World Medical Assembly, Helsinki 1964 and later revisions.

**6.2 CONSENT**

Patients will be identified following discussion at a regional multi-disciplinary team meeting.

Patients will subsequently be seen in the outpatient department by a clinician to discuss their diagnosis, investigation and treatment options. At this stage they will be approached about the study and be provided with a written PINCER patient information leaflet. They will also be provided with a copy of the sub-study SOP.

If patients agree to take part, when they attend for their biopsy or surgery they will be consented for inclusion in the PINCER study by an appropriately trained researcher.

Retrospective consent to access historical FFPE tissue samples & radiology will not be attempted. All samples will be pseudoanonymised, and so the risk to patients is minimal. In addition, the majority of patients will have been discharged from ongoing care but will likely have developed or died from disease recurrence. It is felt that the potential benefit of obtaining retrospective consent significantly outweighs the potential benefit.

Where non-cancerous normal tissue (germline) has undergone genomic analysis, this may identify germline DNA variants and mutations. In cases where an identified germline variant or mutation is to known to increase hereditary cancer risk, this will mandate contact with participant via their general practitioner wherein the participant may choose to undergo genetic counselling.

**6.3 CONFIDENTIALITY**

The Chief Investigator will preserve the confidentiality of participants taking part in the study and will abide by the Data Protection Act.

Samples will be labelled with a randomly allocated study number. The link between patient number and study number will be available only to members of the immediate study team. This link will be recorded on an encrypted password protected spreadsheet stored on NHS computers and will only be available to members of the clinical team involved in the patient’s care. This link is essential to ensure phenotypic & clinical outcome data can be linked to samples.

**6.4 DATA OVERSIGHT**

Each local sub-study lead will be responsible for ensuring compliance with this overarching protocol. These leads will provide local oversight of data collection and storage, as well as compliance with local R&D requirements for tissue storage, transfer and analysis. Responsibilities for tracking and reporting holdings for the University of Liverpool HTA annual return will lie with the sub-study lead. Data sharing with the central study team will ensure overarching governance and be confirmed via regular reviews. This will be part of the steering committee terms of reference (see appendix 3)

**6.5 INDEMNITY**

The University of Liverpool holds Indemnity and insurance cover with Newline Insurance Company, which apply to this study.

**6.6 SPONSOR**

The University of Liverpool will act as the Sponsor for this study. Delegated responsibilities will be assigned to the CI. It is recognised that as an employee of the University the Chief Investigator has been delegated specific duties, as detailed in the Sponsorship Approval Letter.

**6.7 AUDITS**

The study may be subject to inspection and audit by the University of Liverpool under their remit as sponsor and other regulatory bodies to ensure adherence to GCP and the UK Policy Framework for Health and Social Care Research (v3.2 10^th^ October 2017).

**6.8 FUNDING**

Pump priming funding for the platform study will be provided using existing sources (Clatterbridge Novel Research funding grant 2013, £25 000). All sample collection and analysis will be performed by local teams using existing research funding and staff. As such, no further funding is required.

**7. END OF STUDY**

The study will be complete when the recruitment target has been achieved (estimated date 2026).

**8. ARCHIVING**

Data and all appropriate documentation should be stored for a minimum of 5 years after the completion of the study, including the follow‐up period, unless otherwise directed by the funder/sponsor/regulatory bodies.

**9. PUBLICATION POLICY**

All data will be published in peer-reviewed scientific literature.

**10. REFERENCES**

1. Valle J, Wasan H, Palmer DH, Cunningham D, Anthoney A, Maraveyas A, et al. Cisplatin plus gemcitabine versus gemcitabine for biliary tract cancer. N Engl J Med 2010, Apr 8;362(14):1273-81.

2. Hidalgo M, Cascinu S, Kleeff J, Labianca R, Löhr JM, Neoptolemos J, et al. Addressing the challenges of pancreatic cancer: Future directions for improving outcomes. Pancreatology 2015;15(1):8-18.

3. DeOliveira ML, Cunningham SC, Cameron JL, Kamangar F, Winter JM, Lillemoe KD, et al. Cholangiocarcinoma: Thirty-one-year experience with 564 patients at a single institution. Ann Surg 2007, May;245(5):755-62.

4. Ikeyama T, Nagino M, Oda K, Ebata T, Nishio H, Nimura Y. Surgical approach to bismuth type I and II hilar cholangiocarcinomas: Audit of 54 consecutive cases. Ann Surg 2007, Dec;246(6):1052-7.

5. Bliss LA, Witkowski ER, Yang CJ, Tseng JF. Outcomes in operative management of pancreatic cancer. J Surg Oncol 2014, Oct;110(5):592-8.

6. Sirica AE. Cholangiocarcinoma: Molecular targeting strategies for chemoprevention and therapy. Hepatology 2005, Jan;41(1):5-15.

7. Jarnagin WR, Fong Y, DeMatteo RP, Gonen M, Burke EC, Bodniewicz BS J, et al. Staging, resectability, and outcome in 225 patients with hilar cholangiocarcinoma. Ann Surg 2001, Oct;234(4):507-17; discussion 517-9.

8. Ruys AT, Busch OR, Rauws EA, Gouma DJ, van Gulik TM. Prognostic impact of preoperative imaging parameters on resectability of hilar cholangiocarcinoma. HPB Surg 2013;2013:657309.

9. Brutier L, Mastrogiovanni G, Verstegen MM, Francies HE, Gavarro LM, Bradshaw CR et al. Human primary liver derived organoid cultures for disease modeling and drug screening. Nat Med 2017; 23(12):1424-1435.

**Appendix 1;** **PINCER Platform - Sub study Application Form**

Please provide a summary of your sub study. This will be reviewed by the PINCER steering group, and approval given by email. If there are further questions, we will contact you directly.

If you have any questions, please contact robjones@liv.ac.uk.

| Title of Sub study |  |
| --- | --- |
| Lead Applicant |  |
| Lead Applicant email |  |
| Name and email of individual responsible for tissue sample tracking/HTA compliance |  |
|  | |
| Brief aims & objectives of study |  |
| Target patient group |  |
| Anticipated number of patients |  |
| Sample types (number, volume, storage, specific handling processes) |  |
| Location for sample storage (building, room number etc.) |  |
| Anticipated research outputs |  |
| Anticipated start date |  |
| Anticipated duration |  |
| Please confirm MTA is in place to transfer samples from site to University, or your plans to produce an MTA (please attach if relevant) |  |

- I agree to only perform research within the outline of the PINCER protocol, the PINCER favourable ethical opinion, participant consent stipulations and any relevant regulatory frameworks.
- I have valid NIHR GCP certification, and agree to comply with the principles defined therein
  - *Please attach a copy of your certificate*
- I agree to provide 6 monthly updates (or when required) to the PINCER steering group
- I agree to recognise the PINCER team in any outputs from this research
- I have completed human material training (MRC HTA training (https://byglearning.com/mrcrsc-lms/course/index.php?categoryid=1), and agree to comply with the principles defined therein
  - *Please attach a copy of your certificate*
- I agree that samples will not be transferred from the location above to other academic or commercial partners without the formal written approval of the PINCER steering group

Signed:

Name:

Date:

Please send this form or any questions to robjones@liv.ac.uk

**Appendix 2; PINCER Sample Transfer Log**

| **PINCER Study** | **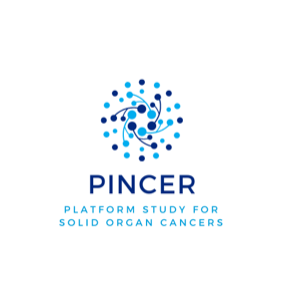** | **REC ID** | 15/NW/0477 |  |  |
| --- | --- | --- | --- | --- | --- |
|  |  | **Version Number:** | 1.0 |  |  |
| Sample Transfer Log |  | **Effective From:** | 1^st^ April 2022 |  |  |
|  |  | **Review Date:** | NA |  |  |

| **Sub-study Title:** | |
| --- | --- |
| **Sub-study Lead Investigator:** | |
| **Tissue collection location:** | **Tissue destination:** |

| **Sample ID** | **Tissue Type and State**  **(e.g. frozen, fresh, slides)** | **Date & Time of Collection/Dispatch** | **Dispatched by**  **(Name & Signature)** | **Date & Time Received** | **Received by**  **(Name & Signature)** |
| --- | --- | --- | --- | --- | --- |
|  |  |  |  |  |  |
|  |  |  |  |  |  |
|  |  |  |  |  |  |
|  |  |  |  |  |  |
|  |  |  |  |  |  |
|  |  |  |  |  |  |
| **Comments:** | | | | | |

| **PINCER STUDY RESPONSIBILITIES** | | **RESEARCHER RESPONSIBILITIES** | |
| --- | --- | --- | --- |
| The study has obtained appropriate consent to retrieve and distribute this tissue as required by the Human Tissue Act (2004) |  | I accept responsibility/custodianship of this tissue |  |
| The study has carefully screened this donor which has been approved as eligible for recruitment |  | As tissue custodian, I agree to appropriately store and maintain accurate records as to the use of this tissue up to and including its disposal. These records must include the study reference number and will be kept for a minimum of 5 years after completion of the study.. I understand I can be audited by the HTA at any time. I will provide reports to the study biannually. |  |
| The study has informed the researcher of all relevant donor and tissue details as permitted by the consent process. In addition, any restrictions or special requirements have also been communicated |  | I will adhere to the University HTA license. I will track and report holdings for the University of Liverpool HTA annual return. |  |
| The study has made every effort to ensure that tissue offered is of appropriate quality, however, donor materials represent living tissue and its characteristics sometimes unavoidably chang e. Tissue is not offered with a warranty |  | I understand that while the donor has been screened, human materials may have hazardous properties, contain infectious agents and pose other health and safety risks |  |
| The study has made every effort to supply tissues according to researcher requirements |  | I understand that donor materials represent living tissues that can change and I will not hold the study responsible for any loss, damage or injury in connection with the use of the donor tissue |  |
| The study has distributed these tissues to projects without favoritism and in accordance with the Steering Committee Terms of Reference (Appendix 3) |  | I agree that tissues will be used within the regulation of the Human Tissue Authority and the specific ethical approval accepted by the study. I will adhere to the UoL HTA license including upon request providing annual returns of the tissues held on premises |  |
|  |  | I will inform the study immediately where there is any adverse event (including relating to the tissue itself, e.g. freezer temperature fluctuations/break donw) or unexpected risk of harm from the receipt or use of the tissue. This will be done by contacting the PINCER steering committee directly |  |
|  |  | I understand that the study can remove access to distributed samples at any time, and I agree to discontinue use of distributed samples if told to do so by the study. |  |
|  |  | I agree to acknowledge the source of the material (PINCER study) in any publication reporting on its use. |  |

**Appendix 3; PINCER Steering Committee Terms of Reference**

**Purpose / role of the committee:**

- To provide oversight for sub-studies run through the PINCER platform
- To assess the scientific rigour and quality of proposed sub-studies
- To ensure sub-studies comply with the protocol, favourable ethical opinion, consent stipulations and any relevant regulatory requirements for the PINCER platform
- To assess applications to transfer samples from the University of Liverpool to other academic and commercial organisations to ensure they comply with the protocol, favourable ethical opnion, consent stipulations and any relevant regulatory requirements forthe PINCER platform

**Membership:**

The committee consists of senior University of Liverpool clinicians and academics. The current membership includes Mr. Robert Jones (Chair), Mr. Dale Vimalachandran, Dr. Ainhoa Mielgo, Prof. Michael Schmid, Prof. Christian Ottensmeier, Prof. Paula Ghaneh, Prof. Christopher Halloran, Prof. Christopher Goldring, Prof. Daniel Palmer.

Membership will likely evolve to reflect changing academic and clinical activity over time. A core membership of 10 will be required for the steering committee. If a member of the steering committee leaves, a replacement will be identified by the remaining steering group and invited to join.

**Working methods**

- Applications for sub-studies will be submitted to the chair
- Applications will be circulated ahead of the steering committee meeting to group members
- Meetings will take place (virtual or face-to-face) on an as-needed basis, not envisaged to be more than 3 times a year
- At least 5 members of the committee will be required to review each application form (see Appendix 1)
- A majority decision will allow adoption onto the PINCER platform
- If a decision cannot be made based on the completed application, queries from the committee about the application will be fed back to the lead applicant. The study may then be adopted via direct chairs approval, or be listed for rediscussion. This decision will be made at the initial steering committee review.

**Review of terms of reference**

These terms of reference will be reviewed by the committee on an annual basis.

Appendix S2

| 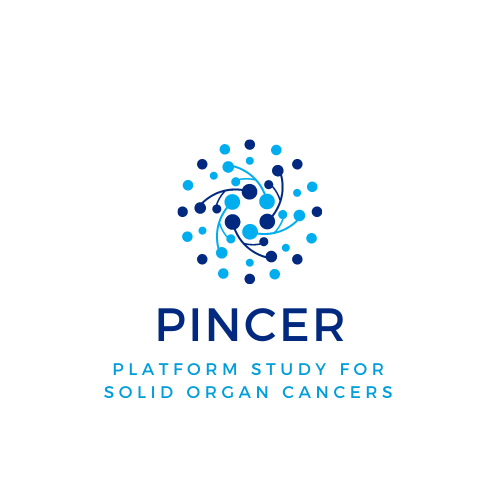 | Standard Operating Procedure | | |
| --- | --- | --- | --- |
|  | SaMPLE COLLECTION AND TISSUE STORAGE FOR PINCER Platform STUDY | | |
| **SOP Number:** | 001 | **Issue Date:** | **4/11/22** |
| **Effective Date:** | 4/11/22 | **Review Date:** | **4/11/23** |

| **Author: (all signatures must be in red ink)** | | |
| --- | --- | --- |
| **Print Name:**  Timothy Gilbert | **Position:**  Associate Principle Investigator | Signature: **Date:** |
| **Approved by: (all signatures must be in red ink)** | | |
| **Print Name:**  Robert Jones | **Position:**  Lead Investigator | **Signature:**  **Date:** |

| **Revision History** | | | |
| --- | --- | --- | --- |
| **Version Number** | **Effective Date** | **Revision Date** | **Reason for Change** |
| 1 |  |  |  |
| 2 |  |  |  |
| 3 |  |  |  |

**Contents**

| **Section** | **Title** |
| --- | --- |
| **1.** | **Applications** |
| **2.** | **background** |
| **3.** | **Purpose** |
| **4.** | **SCOPE** |
| **5.** | **Procedure** |
| *5.1* | ***Obtaining informed consent*** |
| *5.2* | ***Blood sample collection*** |
| *5.4* | ***Surgical sample collection*** |
| *5.5* | ***Tissue biopsy collection*** |
| **6.** | **RESPONSIBILITIES** |
| **7.** | **APPENDIX - workflow** |
|  |  |

1. **RELEVANCE**

This standard operating procedure (SOP) is relevant to competent research personnel who are delegated to collect samples from patients enrolled in the PINCER platform study. This document also applies to research staff responsible for the processing and storage of these collected tissues in laboratory facilities.

1. **BACKGROUND**

The collection of human tissue is governed by the Human Tissue Act (2004) and EU directives on research governance. It is the responsibility of everyone to have read and understood the relevant documentation in order to abide by the regulations.

The Institute of systems, molecular and integrative biology (ISMIB, UoL) supports the collection and storage of tissue from solid cancers for translational research. Researchers are required to participate in the collection and storage of samples for ethically approved research as part of the PINCER study platform. The systematic and regular collection of both benign and malignant tissue is fundamental to these research activities. The process of tissue collection requires a co-ordinated approach from clinicians and research staff and encompasses the process of patient consent, collection, processing, and storage of tissue samples from patients with solid cancers.

1. **PURPOSE**

This SOP has been written to provide uniform procedures for the collection of blood, and tissue samples as well as other relevant biological specimens from patients with solid cancers for research use. Any issues relating to tissue collection should be referred directly to Mr Robert Jones (Lead Investigator for the PINCER Platform) who should also be informed of all SOP deviations with respect to the samples.

1. **SCOPE**

This SOP applies to all researchers collecting, processing and storing samples collected as part of the PINCER platform study.

**5. PROCEDURE**

**5.1 Identification of Eligible Patients**

- It is the responsibility of the researchers requiring patient samples to liaise with the clinical team regarding upcoming cases that involve potentially eligible patients
- Following identification of these patients it is the responsibility of the researchers to liaise with clinical members of staff to ensure a suitability trained individual is available to consent and collect clinical sample
- Following identification of an eligible patient permission should be sought from that patient’s responsible consultant to ensure they are happy for research samples to be collected on their patient

**5.2 Obtaining Informed Consent:**

- **Consent for specimen collection must be taken prior to tissue collection** and must be performed by a **suitably trained Doctor, Nurse or Research Practioner who is on the PINCER delegation log**. They will approach the patient, provide a Patient Information Sheet and take informed consent.
- It is anticipated that all patients scheduled to undergo surgery for a solid cancer will be given the opportunity to take part in the PINCER research programme. It should be an informed, non-coercive discussion in appropriate language that will be understood by the patient.
- Informed consent must be undertaken prior to theatre and prior to any medications being administered which may alter the participants ability to give consent.
- As part of the consent process the patient should be given the opportunity to ask questions about the project, understand why the research is being done and any foreseeable risks involved.
- Opportunity should be given for patient’s questions and answers provided. Where an answer can’t be provided this should be sought from someone able to do so.
- The patient should be given ample time to read the Patient Information Sheet relating to the research before the consent form is signed.
- It should be explained to the patient that participation is voluntary and that they may withdraw at any time from the project, without giving any reason and without medical care or legal rights being affected.
- The participant should be aware that there are no financial benefits if the research leads to the development of new treatments or medical tests.

1. **Once the patient has agreed to specimen collection they must initial each specific point as detailed consent form in the adjacent boxes on the form indicating their understanding. Then both researcher and patient sign and date the consent form.** **The Researcher taking consent must ensure that the form is correctly filled out with participants initials in all the correct boxes.**
2. **The original signed consent form should be placed in the PINCER site file (9^th^ floor office research cupboard– RLUH), a copy should be given to the patient and a copy should be placed within the patient’s case notes. A written note should also be added to the patient electronic case note record (PENS) stating their agreement to participate.**

- Once consent has been taken, the researcher must inform the operating surgeon/responsible clinician that the patient has consented for tissue collection.
- It is the researcher’s responsibility to ensure that individuals physically taking the samples are happy with the individual sampling techniques/number of specimens required. The amount of sample taken (surgical sample/biopsy) remains at the discretion of treating clinician (surgeon/radiologist/endoscopist) within the constraints of the over-arching research protocol
- The mobile number for the researcher must be made available to clinical staff such that they can be contacted prior to sample collection and in case of any issues.

**5.3. Blood Sample Collection:**

1. **Informed consent must be obtained** from the patient prior to any samples being taken as per section 5.2.
2. Sample must be collected by a **suitably trained clinician (doctor, nurse, phlebotomist)** ideally this individual should be directly involved with the specific study being undertaken
3. Blood tubes and volumes of blood required will vary according to the individual project and should be collected in line with their specific study SOP. Where the suitably trained individual is not directly involved in the study it the responsibility of the researcher requesting specimen collection to ensure that the correct blood bottles and collecting kit are provided and that bottles are correctly labelled.
4. It may be appropriate for the patient to have blood samples taken while under general anaesthetic – where this is appropriate samples should be taken in coordination with the anaesthetic team and only when they are happy to do so. In this situation the clinician taking samples from an anaesthetised patient must be shown the sign consent form.
5. Once the sample has been obtained from the participant the researcher must then collect the sample and the arrange for transfer of this specimen to the University for further processing or storage as required. All processing and storage should be performed in appropriate facilities as governed by the Human Tissue Act (2004).

**5.4 Surgical sample collection (theatre):**

1. **Informed consent must be obtained** from the patient prior to any samples being taken as per section 5.2
2. 30 mins prior to removal of the surgical specimen the researcher collecting the samples will receive the call to theatre**,** they should bring with them the required cryovials and relevant buffers for the storage of the samples, along with a secure

container containing ice for transfer to the University.

1. On arrival to the theatre complex the researcher must change into appropriate theatre attire. Where the researcher is a clinical member of staff with theatre access they can proceed directly to the required theatre. Where the researcher is from the university and not at a member of hospital staff they must sign in at reception and then wait to be shown to the correct theatre.
2. Once the specimen is resected sampling can then be undertaken. The researcher sampling the specimen must confirm with the operating consultant that they remain happy for the specimen to be sampled.
3. Surgical instruments may be required to enable sampling and marking of the resection margins (e.g. blade/forceps/sutures). **If such items are used they must be done so in accordance with wishes of the scrub nurse and circulating team members**. This is to ensure none of these items interfere with, or are missed off, the final scrub count.
4. Sampling of the resected specimen **should only be done by a clinician** with sufficient experience to ensure that there remains **sufficient tissue for histopathology** with minimal disruption to the relevant resection margins. Sampling should not be performed directly through the resection margin which will be used to assess surgical clearance. For example, colorectal cancer should be sampled by opening the colon cleanly and sampling from inside the lumen. Liver resection should be sampled by slicing the liver from the capsular surface, and sampling within the liver substance. Once taken, samples should be put within labelled cryovials and placed on ice (provided by the researcher). A photo can be taken as needed (digital camera, smart phone or similar) to allow for localisation of samples in specimen.
5. Once the specimen has been sampled the remaining specimen can then be potted for histopathology. The **histopathology form must be updated by the sampling clinician to detail where and how the specimen was sampled.**
6. Once the above steps have been completed the research samples can then be transferred for further processing and storage. All processing and storage should be performed in appropriate facilities as governed by the Human Tissue Act (2004).

**5.5 Tissue biopsy collection (IR/endoscopy):**

1. **Informed consent must be obtained** from the patient prior to any samples being taken as per section 5.2
2. 30 mins prior to removal of the specimen the researcher collecting the samples will receive the call to endoscopy/radiology**,** they should bring with them the required cryovials for the storage of the samples, along with a secure

container containing ice for transfer to the University.

1. On arrival to the endoscopy or radiology suites the researcher must change into appropriate attire. Where the researcher is a clinical member of staff with theatre access they can proceed directly to the required theatre. Where the researcher is from the university and not at a member of hospital staff they must sign in at reception and then wait to be shown to the correct room.
2. Sampling of the resected specimen **should only be done by a member of the clinical team** with sufficient experience to ensure that there remains **sufficient tissue for histopathology**. Once taken samples should be put within labelled cryovials and placed on ice (provided by the researcher).
3. Once the above steps have been completed the research samples can then be transferred to the university for further processing and storage. All processing and storage should be performed in appropriate facilities as governed by the Human Tissue Act (2004).

**6.0 REPSONSIBILITY**

**5.1.1 Responsibilities of the Researcher:**

It is the responsibility of the researcher to:

- Initiate contact with clinical staff to check for eligible patients.
- Ensure a suitably trained individual is available to obtain informed consent prior to tissue collection and that consent has been taken prior to specimen collection
- Ensure that individuals physically taking the samples are happy with the individual sampling techniques/number of specimens required. Understand the amount of sample taken (surgical sample/biopsy) remains at the discretion of treating clinician (surgeon/radiologist/endoscopist).
- Check with an appropriate member of theatre/endoscopy/radiology staff whether the case is going ahead.

- Provide the necessary for kit for storage and transfer of the samples to the university
- Ensure that relevant clinical staff are aware how they can be contacted immediately.
- Ensures that all obtained samples are processed and stored in appropriate facilities within the university as governed by the Human Tissue Act (2004) and ensure that they maintain an anonymised electronic record of sample held.

**5.1.2 Responsibilities of the Clinical team:**

It is the responsibility of the clinical team:

- Work constructively with the research team to help identify eligible patients.
- Take informed consent as detailed in section 5.2. Ensure copies of the consent form are placed within patient notes and original within the site file.
- To work constructively with the research team to ensure they understand the sampling requirements.
- Obtain blood and tissue samples from the patient as outlined in this SOP. Ensuring that consent has been taken prior to sampling
- Ensure that where tissue samples are taken (surgical/biopsy) there remains **sufficient tissue for histopathology** with minimal disruption to the relevant resection margins. The amount of sample taken (surgical sample/biopsy) remains at their discretion with ultimate responsibility lying with consultant in charge.
- To contact the researcher 30mins prior to specimen being removed from the patient
- To contact the researcher in the event the case fails to go ahead, the specimen is subsequently deemed unsuitable for sampling or the patient refuses/withdraws consent.

**Appendix 1: Workflow**

The researcher transfers samples to the university and ensures and further processing/storage is done so in accordance with HTA requirements. Researcher retains responsibility for maintaining an electronic record to specimens held as required for HTA returns

Clinical team collect sample in accordance the guidelines detailed in this SOP and ensures histopathology form is updated Samples are then given to the researcher for transfer to the university.

Researcher receives a phone call/secure WhatsApp message 30mins prior to sample collection. Researcher brings with them the required kit to collect store and transfer samples to university

The clinical team confirm that the patient has agreed to specimen collection and place consent forms in clinical notes and site file. Researcher and clinical team liaise to ensure sampling requirement are understood

The researcher and clinical team liaise on the day prior to specimen collection to ensure that an appropriate individual is available to consent the patient

Researcher liaises by a secure WhatsApp group with the clinical team regarding upcoming cases with potentially eligible patients.
